# To Share or Not to Share? A Survey of Biomedical Researchers in the U.S. Southwest, an Ethnically Diverse Region

**DOI:** 10.1371/journal.pone.0138239

**Published:** 2015-09-17

**Authors:** Mai H. Oushy, Rebecca Palacios, Alan E. C. Holden, Amelie G. Ramirez, Kipling J. Gallion, Mary A. O’Connell

**Affiliations:** 1 Plant and Environmental Sciences, New Mexico State University, Las Cruces, New Mexico, United States of America; 2 Public Health Sciences, New Mexico State University, Las Cruces, New Mexico, United States of America; 3 Institute for Health Promotion Research, University of Texas Health Science Center, San Antonio, Texas, United States of America; Renal Division, Peking University First Hospital, CHINA

## Abstract

**Background:**

Cancer health disparities research depends on access to biospecimens from diverse racial/ethnic populations. This multimethodological study, using mixed methods for quantitative and qualitative analysis of survey results, assessed barriers, concerns, and practices for sharing biospecimens/data among researchers working with biospecimens from minority populations in a 5 state region of the United States (Arizona, Colorado, New Mexico, Oklahoma, and Texas). The ultimate goals of this research were to understand data sharing barriers among biomedical researchers; guide strategies to increase participation in biospecimen research; and strengthen collaborative opportunities among researchers.

**Methods and Population:**

Email invitations to anonymous participants (n = 605 individuals identified by the NIH RePORT database), resulted in 112 responses. The survey assessed demographics, specimen collection data, and attitudes about virtual biorepositories. Respondents were primarily principal investigators at PhD granting institutions (91.1%) conducting basic (62.3%) research; most were non-Hispanic White (63.4%) and men (60.6%). The low response rate limited the statistical power of the analyses, further the number of respondents for each survey question was variable.

**Results:**

Findings from this study identified barriers to biospecimen research, including lack of access to sufficient biospecimens, and limited availability of diverse tissue samples. Many of these barriers can be attributed to poor annotation of biospecimens, and researchers’ unwillingness to share existing collections. Addressing these barriers to accessing biospecimens is essential to combating cancer in general and cancer health disparities in particular. This study confirmed researchers’ willingness to participate in a virtual biorepository (n = 50 respondents agreed). However, researchers in this region listed clear specifications for establishing and using such a biorepository: specifications related to standardized procedures, funding, and protections of human subjects and intellectual property. The results help guide strategies to increase data sharing behaviors and to increase participation of researchers with multiethnic biospecimen collections in collaborative research endeavors

**Conclusions:**

Data sharing by researchers is essential to leveraging knowledge and resources needed for the advancement of research on cancer health disparities. Although U.S. funding entities have guidelines for data and resource sharing, future efforts should address researcher preferences in order to promote collaboration to address cancer health disparities.

## Introduction

Understanding, overcoming and ultimately eliminating cancer health disparities is a major public health objective. Recent advances in areas such as cancer gene expression, epigenetics, and proteomics, hold great promise for reducing such disparities. These advances include improved risk prediction, early detection, more precise diagnosis and prognosis estimation as well as targeted therapeutics as part of personalized and precision medicine [1]. Biobanks or biospecimen repositories are essential in serving the needs of personalized medicine and genomic research [2] as they provide the sample sets (e.g., tumor tissue, blood, urine) and related electronic medical/health information for this research [3]. There are many challenges to sharing both the data and the specimens themselves, between labs at one institution, between institutions in one country and between countries [4–7]. In contrast to the benefits of data sharing which are well described and understood, individual researchers still perceive risks associated with data sharing and may be reluctant to comply with requirements for sharing, reviewed in [8]. Understanding the concerns of researchers for data and biospecimen sharing are essential if we are to develop policies that both reward sharing and reduce the risk of sharing as perceived by the individual researcher. Further, inclusion of biospecimen samples that represent the full range of diversity among humans in research activities is essential if health disparities are to be eliminated. Sharing those samples and/or the data from their analysis among health disparities researchers is likely to be the most efficient method [6] to include members of diverse populations in research advances. A clear understanding of the perceptions among researchers of how they prefer to share their data and samples is needed.

### Biorepositories in cancer research

In cancer research, the increasing demand for specific, highly annotated human cancer biospecimens underscores the importance of biobanks [9,10]. An ultimate goal of biorepositories would be to provide access to well-annotated biospecimen collections from diverse populations in order to address cancer health disparities in the context of personalized medicine.

Unfortunately, there is a dearth of high quality, well-annotated and accessible cancer biospecimens [11–13]. This is due in part to the decentralized and ad hoc nature of the current system for collecting and maintaining biospecimens, which has resulted in collections with narrow scope (i.e. limited cancer types), inefficient processes for accessing samples, limited access and utility of existing samples, and inadequately stored biospecimens [3]. Furthermore, the annotation process is complex and expensive and each tissue bank may define and apply annotation differently [10]. Cancer researchers, therefore, face several challenges in identifying and obtaining sufficient, well annotated, high-quality tumor tissues for their research programs [10].

Cancer genomic studies not only require high-quality samples, but they require adequate numbers of samples as well [11]. Unfortunately, researchers in the Cancer Human Biobank (caHUB) study [14] reported difficulty obtaining adequate numbers of biospecimens, with 69% of their sample reporting “some” degree of difficulty in accessing the quantity of biospecimens needed for their research. One way of increasing the number of samples available for such studies is for tumor banks to collaborate and pool their resources. This could be achieved by establishing a central collection to which tumor banks contribute; or by forming networks where multiple tumor banks share a common online database, but retain control of their collections [7,11,15]. Creating a virtual repository or database that uses a common standardized informatics platform for identifying human tissue samples located in multiple sites, can facilitate the identification of cancer-specific biomarkers and encourage collaborative research efforts among researchers [6,10]. Moreover, virtual biobanks create a central resource through which researchers can find highly annotated tissue samples/data, and access a larger specimen collection than that available at an individual institution [16].

Several U.S. efforts were undertaken to address the critical and problematic shortage of high quality, well-documented biospecimens for cancer research including the caHUB and The Cancer Genome Atlas (TCGA). The caHUB is an open access, standardized human biospecimen resource http://biospecimens.cancer.gov/programs/cahub/default.asp, whereas TCGA conducts comprehensive genomic characterization and analysis of many distinct and important cancer types http://cancergenome.nih.gov/. The comprehensive data generated by TCGA’s network are freely available and widely used by the cancer community through the TCGA Data Portal (URL, cited above. In addition to these initiatives, several U.S. funding sources now have existing policies supporting or mandating biospecimen data sharing, most notably the National Institutes of Health [17]. These policies expand and underscore the importance of data sharing usually required by publication policies of major biomedical journals [18,19]

### Obstacles and risks for biospecimen sharing

Despite efforts to increase data sharing and mandate full access to research/trial data [20,21], investigators who rely on human subject participation are among the least likely to share their raw data [22,23]. One reason may be that biomedical researchers and investigators spend substantial time, money and effort collecting data and specimens for research and clinical trials [15]. The rigorous nature and cost of this work results in researchers’ developing a sense of ownership to the data and samples collected and a desire to preserve exclusive rights to data that has taken many years to produce [24]. Additional obstacles to data sharing may include resources required for long-term data management, lack of rewards or recognition for conducting original research, control over data access, and funding [24–28]. There have been efforts to address the lack of recognition of the role of biorepositories in advancing health science research. Several international groups have started to work with scientific journals to develop a publication policy that will require the inclusion of a specific ID reflecting the biospecimens included in the published study. This will then allow the biorepositories to report on the utility of their resources as reflected in a publication stream using the BRIF (Bioresource Research Impact Factor) [4,29–31].

#### Informed consent issues in biobanking

In addition to researchers’ obstacles to sharing biospecimen resources, past studies have also identified several concerns on biobanking by prospective donors. Subject confidentiality and privacy, as well as inappropriate secondary use of biospecimen data were frequently reported issues of concern by prospective biorepository participants [32–34]. Moreover, prospective donors reported concerns regarding the informed consent process (e.g., lack of association with a specific study purpose). Hesitation among prospective donors to consent to future use of their leftover biosamples raises issues on the type of consent forms researchers should develop. Whereas a research-specific consent process limits the use of biospecimens for very specific and pre-defined research purposes, a general-purpose consent process allows for a broader array of research purposes and the potential for future collaborative research [5,32]. In addition to these two types there are also tiered consents which allow the donor to consent to a menu of potential future uses of their samples, and the option re-contact to request future permissions [35]. The use of a global or general informed consent form is common and often preferred by researchers, but this type of consent may not be fully understood by particular minority groups, as evidenced by the case of Arizona State University vs. the Havasupai Tribe [36]. In this particular case the tribe approved studies for type II diabetes, but were unaware of objectionable uses by researchers for studies of schizophrenia, inbreeding and evolutionary genetics [35]. The more complex informed consent processes, i.e. specific uses and tiered consents, place additional requirements on the researcher to ensure that there are not inappropriate secondary uses of the samples or the data.

### Diversity issues in biobanking

The U.S. NIH National Cancer Institute-Center to Reduce Cancer Health Disparities (NCI-CRCHD) launched the Minority Biospecimen and Biobanking Program (BMaP) in 2009 to address the lack of diverse biospecimen resources for cancer research [37]. BMaP aimed to create networks/centers to ensure the adequate and continuous supply of high quality human biospecimens from multi-ethnic communities for cancer research in six geographic regions. One of the geographically largest and most diverse BMaP regions was Region 4, which encompassed Arizona (AZ), Colorado (CO), New Mexico (NM), Oklahoma (OK), and Texas (TX). Hispanics represent 46.3, 37.6, 29.6, 20.7, and 8.9% of the state populations respectively in NM, TX, AZ, CO and OK; while Native Americans represent 12.9, 10.7, 5.5, 2.1 and 0.4% respectively in OK, NM, AZ, CO and TX based on the U.S. Census Bureau, 2010 Census (http://factfinder.census.gov/faces/nav/jsf/pages/index.xhtml).

Cancer health disparities are major health issues among racial, ethnic, and underserved groups in this southwestern region. Hispanics have higher cancer incidences rates for hepatocellular cancer [38,39], and gastric cancer [40]. Hispanic women in some cases have higher mortality rates for breast cancer that includes some hormone responsive tumor types [41,42]. Testicular cancer is increasing much more rapidly among Hispanic adolescents than in non-Hispanic white teenagers [43]. The role of racial and ethnic differences in the biology of specific cancers is predicted to explain some cancer health disparities outcomes, along with cultural, environmental, and socioeconomic factors [44]. In order to develop treatments that target specific cancer biological types, or cancers that may be more prevalent among some minority populations, researchers will require access to biospecimens from diverse populations.

In addition to all the above-mentioned barriers, researchers report limited availability of diverse tissue samples [11,45,46]. Two major issues in this regard are insufficient collection of diverse samples and lack of annotation capturing such diversity within existing biospecimen pools. To date, most biorepositories have low representation of samples from minority populations [45–48]. Researchers working in areas with diverse populations can play a role in increasing the diversity of available biospecimen samples. Targeting collection rates that effectively reflect a region’s demographics [9] can promote diversity in biospecimen pools. However, this may be easier said than done. A recent report by another regional BMaP network, Region 5 (15 states in the Midwestern and Northeastern U.S.) [49], indicated that within the subset of biospecimen collections with ethnicity annotations, only 10% were from minority populations; and within a larger group of specimen collections with racial annotations, only 11% were from non-white donors. These descriptions of biospecimens were obtained following detailed phone interviews with the administrative leads of 10 biorepositories within that region.

Many of the concerns listed above (e.g., privacy/security, misuse of data) by prospective donors are even more pervasive among minority groups [32–34,46,50]. An additional barrier to biospecimen donation among racial/ethnic minority groups includes distrust of the health care system [51,52]. Misuse of biospecimens collected from the Havasupai Tribe in Arizona is a recent example of the basis for the mistrust racial and ethnic groups may hold against biomedical research [35,52]. Finally, some prospective donors from diverse populations reported they were not even aware of clinical trials and related opportunities for donating biospecimens [33,51].

Previous research on sharing of biospecimens/biospecimen data has focused on data sharing-related needs, perceptions and practices of researchers participating in different settings, including clinical trials, academic/medical institutions, and institutional review boards [15,18,20,21,26,53–55]. However, little to none of this research has addressed data sharing attitudes and practices by researchers who actually have access to and work with biospecimen samples from ethnically diverse populations. Understanding the data sharing needs and concerns of investigators working with biospecimens collected from racial/ethnic minority groups is essential to increasing the diversity of samples available for cancer health disparities research [45,46].

The purpose of the present study was to assess barriers, concerns, and practices for sharing biospecimens and related data among researchers in BMaP Region 4. We chose this region for study as this region included our home institutions and we were charged by the NIH to investigate these barriers within our home BMaP region. Because the study included researchers who collect biospecimens from minority populations, it may help elucidate barriers to increasing the diversity of samples within central biobanks/biorepositories. The ultimate goals of this research were to understand data sharing barriers among biomedical researchers; guide strategies to increase participation in research utilizing high quality, multiethnic human biospecimens; and strengthen collaborative opportunities among researchers in this region. Finally, although previous studies have largely examined potential donors’ reasons for refusing to donate [34,46,50], the present study examined researcher’s ‘perceived’ reasons why individuals refuse to donate.

## Materials and Methods

### Rationale for approach

We surveyed NIH funded investigators working in our geographical region on their attitudes about sharing biospecimens. We chose this population because we were aware that the collections of biospecimens in academia were highly distributed. This observation was based on a regional inventory of biospecimen resources we had conducted as part of our activities as a BMaP network for NCI CRCHD. There was no single point of contact at any one institution in our region for the management of all of the human biospecimens at that institution. Rather, specific researchers designed protocols and experiments to collect the samples necessary for their research projects. Some of these samples, but not all of them, might be stored in a centralized facility administered at an institutional level.

Our choice of participants was further driven by our interest in determining from the researcher’s perspective the factors that influenced research using ethnically and racially diverse human biospecimens. This level of response was missing from the literature, there were a few reports polling biorepository directors [5,49,56,57] but virtually nothing surveying principal investigators/researchers. Our survey was therefore designed to be short and administered via an electronic web interface, as we recruited participants from an NIH database of funded investigators (see below). No specific examples of data types were provided with the survey, the questions were therefore open to interpretation by the respondent. The survey was developed following ~18 months of teleconferences and several face-to-face meetings at both a regional and national level of assemblies of BMaP participants. The language and questions in the survey were informed by those discussions.

### Ethics statement

The New Mexico State University Human Subjects Institutional Review Board approved this study (FWA00000451; NMSU IRB #490) as an online survey (SurveyMonkey®), which protected participants’ confidentiality and offered complete anonymity. A PDF version of the electronic survey tool including the informed consent request is provided as S1 Survey Tool–Researchers' Attitudes to Participation in a Virtual Biorepository. Individuals self-identified their age, gender, race and ethnicity using menu options in the electronic survey tool. No identifying information (name, institution, responding email, or IP address of responding computer) was recorded.

### Study design and population

The population for this study included federally funded researchers in Region 4 states who had worked directly or indirectly with human biospecimens in the last 5 years. For this multimethodological study, using mixed methods for quantitative and qualitative analysis of survey results, the research team used purposive, non-probability sampling to identify and recruit participants using a publically available NIH database. The search on NIH RePORT database used the key terms, human biospecimen research, human tissue research, tissue collection, cancer research, human cell line research, cancer health disparities research, and human tissue research; and identified 689 individual researchers. Researchers who were not federally funded, not in any of the Region 4 states, and not working in projects directly or indirectly related to human biospecimens were excluded from the search and therefore from the study. Participants were invited to participate via their email addresses, which were obtained from the NIH RePORT database.

### Participant recruitment

Invitations (n = 689) to identified participants in state-based cohorts were sent by email December 2011 through March 2012. The email contained a description of the study, an embedded informed consent statement, along with a secure link to access the anonymous protected online survey. Two follow up reminders were sent two weeks apart, for a total of three notifications. A small number of email addresses were invalid, 84 (12.2%); of the remaining 605 presumably valid invitations, we received 112 responses (18.5%). This response rate, while low is similar to the response rate of a similar study examining NIH funded researchers’ attitudes about caHUB; in that instance the response rate was 14% [14,58]. The state specific response rates varied; the highest response rates were in AZ (26 respondents /109 email invitations) 24% and NM, (32/134) 24%, followed by CO, (19/98) 19%, while TX, (28/206) 14%, and OK, (7/58) 12% were the lowest.

### Research instrument

A survey instrument was developed to examine researchers’ perceptions and willingness to participate in a virtual biorepository, as well as their perceived attitudes and barriers to sharing data in such a biorepository. The survey consisted of 24 items comprised of 18 yes/no and multiple-choice questions, 2 questions with Likert–type response scales (5-point; ranging from “very likely” to “very unlikely”), and four open-ended questions. They were grouped into two main sections: 1) researcher demographics (age, gender, ethnicity, professional rank, and degree(s) granted by the host institution), and 2) beliefs regarding data sharing through a virtual national biospecimen database. The second section captured the researcher’s biospecimen collection practices, perceived barriers and criteria for participating in a virtual national biospecimen database, and perceived barriers to biospecimen donations among the general population. Respondents were also asked about their type of research work, collection practices for human tissues/biospecimens within the past 5 years, percentage of tissue collection from diverse populations, type of informed consent and preferences for components of a virtual biorepository. Researchers were also asked whether or not they agree with the NIH Resource Sharing Plan. Additionally, two questions assessed whether researchers would obtain samples for their research work or share data about biospecimens they have collected through a virtual national biorepository. Additional questions asked about the type of information researchers would agree to share about themselves, their research and data, and their tissue collection.

Finally, open-ended questions assessed respondents’ perceived barriers to implementation of a virtual national biorepository: perceived reasons why individuals refuse to donate specimens, personal requirements for collaborating and sharing specimens with other investigators, and their major concerns if unwilling to share specimens. SurveyMonkey®, an online survey software tool, was used for administering the questionnaire and for data collection. The entire survey is presented as S1 Survey Tool–Researchers' Attitudes to Participation in a Virtual Biorepository).

### Statistical methods–quantitative data

Quantitative data in this study were analyzed using IBM SPSS Statistics version 19.0 (SPSS Inc., Chicago Ill. 2010). Chi-square, Factor Analysis and One-Way ANOVA were utilized. Primary analyses evaluated associations between participant demographic characteristics and participant attitudes towards and intentions to participate in a virtual national biorepository. Secondary analyses examined relationships between participant professional characteristics and the same set of dependent variables.

Next, we employed principal components analysis (PCA) and factor-analyzed the 14 items describing the conditions under which respondents felt they would participate in the database and specific aspects of that participation [59]. Latent structural and psychometric validation analyses were conducted in accordance with guides on sample sizes for factor analysis, after assessing the suitability of this data set for dimensionality analysis [60–62]. To this end we examined the correlation matrix of candidate measures utilizing various criteria (e.g. most of the 14 items in the correlation matrix had inter-correlations of 0.30 and above). Kaiser-Meyer-Olkin (KMO) measure of sampling adequacy was 0.74, indicating the partial correlations among matrix variables were smaller (less inter-correlated) than the recommended value of 0.60 [63,64]. Bartlett’s Test of Sphericity (an indicator that the identity matrix is appropriate for factor analysis), returned a value of p = 0.03. These two tests encouraged the appropriateness of dimensionality analyses of the correlation matrix [63–65].

The initial un-rotated PCA matrix revealed four components with eigenvalues >1 (the generally accepted criterion for identification of “important components”) [59,61,62], explaining a total of 65.6% of the variance. Assessment of the scree plot (a distributional “map” of items associated with factors) showed three factors were psychometrically meaningful. Cattell’s Scree Plot coefficient [66], combined with the item-loadings on factors and their associated eigenvalues indicated that three components should be retained for further analysis. The components matrix revealed that six items “loaded” (correlated highly with) on the first factor, three loaded on the second factor, and three loaded on the third factor. Table 1 lists the survey questions or components that loaded on these factors. One item only described the fourth factor and it was discarded. Similarly, one item loaded significantly on no factor and was also discarded. This left a set of three component factors, which together explained 60.6% of the variance in the item-pool correlation matrix. VARIMAX Rotation (a manipulation of factors to maximize correlation of individual items and the factor) produced three potential scales given in Table 1.

**Table 1:** Factor Scales and Associated Items (Would you agree to publish…in an NIH funded Region 4 database?).

	Factor Components		
Factors and Related Scale Items	1	2	3
**Scale I: Share Specimen Information, *alpha* = 0.835**			
Q19h: the tissue/sample types of your data collection…?	.857	-	-
Q19b: the number of specimens in your tissue collection …?	.800	-	-
Q19g: the methods of sample collection in your tissue collection…?	.707	-	-
Q19f: the clinical diagnosis of specimen donors in your tissue collection …?	.599	-	-
Q19a: data about your tissues …?	.497	-	-
Q20b: PI name and contact information …?	.322	-	-
**Scale II: Share Donor Information, *alpha* = 0.869**			
Q19d: the gender of specimen donors in your tissue collection …?	-	.853	-
Q19c: the age of specimen donors in your tissue collection …?	-	.853	-
Q19e: the ethnicity of specimen donors in your tissue collection …?	-	.723	-
**Scale III: Share Grant Information, *alpha* = 0.807**			
Q20f: your source of funding …?	-	-	.856
Q20e: your grant abstract …?	-	-	.837
Q20d: link to publications of yours and your research …?	-	-	.803

Extraction Method: Principal Component Analysis. Rotation Method: Varimax with Kaiser Normalization.

Principal component analysis detected three factor based scalar constructs (Table 1): “SI Share Specimen Information”, “SII Share Donor Information”, and “SIII Share Grant Information”. Factors were evaluated contextually and reliability tests determined the degree to which items of the resultant scales represented a coherent set of items measuring a targeted underlying construct [67,68]. Cronbach *coefficient alpha* values for the scales were all greater than 0.800 (Table 1).

### Qualitative data analysis

We conducted a descriptive thematic analysis of the open-ended response questions using two independent raters. Specifically, a content analysis technique identified emergent themes from the responses to each question. The final coding categories (i.e., response themes) were established and coded by two separate researchers for all participant responses. Inconsistencies across the independent raters were resolved using a third rater. Response frequencies and percentages were then calculated for each response theme (i.e., code) within each question for the overall sample. The frequencies of these response themes were also sorted based on the self-identified ethnicity of the respondent or on the gender of the respondent. All of the verbatim de-identified responses to the four open-ended survey questions are provided in S1 Dataset. The method of participant selection, data analysis and data reporting are compliant with current guidelines for qualitative data analysis [69].

### Limitations

One limitation in this study was the low response rate (18.5%). Electronic surveys of researchers, however, historically have had low response rates; 9% and 14% have been previously reported [14,58]. This low response rate has limited the statistical power in the study to detect group differences. Additionally, the sampling design was purposively non-random with participant selection based on funding and/or activity expressed in the NIH RePORT. This may have limited the identification of additional qualifying participants due to lack of up-to-date detailed information in the NIH RePORT database. The number of researchers responding to each question in the survey was variable and thus the “relative weight” of an answer is different question to question; we have provided the frequency of response for each question to inform the reader. Finally, given the convenience sample used in this study, respondents may not fully represent the researcher population in Region 4.

## Results

The purpose of this study was to assess biospecimen and related sample data sharing beliefs and practices and perceived barriers to implementation of a biorepository among researchers in the largely diverse BMaP Region 4. This section will describe participant characteristics, current practices and barriers in biospecimen research, and biospecimen and related sample data sharing preferences and needed resources for establishment of a virtual national biorepository.

### Characteristics of participants

Table 2 provides the self-reported characteristics of the participants. The majority of respondents self-identified as non-Hispanic white (n = 64, 57.1% of total), and male (n = 63, 56.2% of total); most respondents were either in the 40–49 age group (n = 42, 37.5% of total), or the 50–59 age group (n = 31, 27.2%). The overwhelming majority identified as principal investigators (n = 98, 87.5% of total) working at PhD granting institutions (n = 100, 89.3%). The majority engaged in basic (n = 69, 61.6%), or translational (n = 50, 44.6%) research, with only a few respondents engaging in clinical or epidemiological research; some respondents selected more than one category for their primary research type. The states in Region 4 were nearly equally represented with the exception of OK which was poorly represented in this study (6.3%).

**Table 2:** Researcher participant characteristics.

Characteristic	Sub-characteristic	# of participants (% respondents)	% of 112 total participants
Age (# of respondents who supplied their age = 103)	<29	3 (2.9^a^)	2.7
	30–39	8 (7.8)	7.1
	40–49	42 (40.8)	37.5
	50–59	31 (30.1)	27.7
	60–69	14 (13.6)	12.5
	> 70	5 (4.9)	4.5
Gender (# of respondents who supplied their gender = 104)	Male	63 (60.6)	56.2
	Female	41 (39.4)	36.6
Race/Ethnicity (# of respondents who supplied their race = 101)	White (non-Hispanic)	64 (63.4)	57.1
	Hispanic	18 (17.8)	16.1
	Asian	14 (13.9)	12.5
	Other^b^	5 (5.0)	4.5
Location (USA State) (# of respondents who supplied their location = 112)	Arizona	26 (23.2)	23.2
	Colorado	19 (17.0)	17.0
	New Mexico	32 (28.6)	28.6
	Oklahoma	7 (6.3)	6.3
	Texas	28 (25.0)	25.0
Role in Research (# of respondents who supplied their role = 111)	Principal Investigator	98 (88.3)	87.5
	Other	13 (11.7)	11.6
Research Institution (# of respondents who supplied their institutional level = 110)	PhD granting Inst.	100 (90.9)	89.3
	other	10 (9.1)	8.9
Type of Researcher^c^ (# of respondents who supplied their type of research = 112)	Basic	69 (61.6)	61.6
	Translational	50 (44.6)	44.6
	Clinical	10 (8.9)	8.9
	Epidemiological	6 (5.4)	5.4

^a^Sub-characteristic percentage calculated as number of participants with sub-characteristic/number of respondents for that sub-characteristic, eg. 3/103 x 100 = 2.9%

^b^Includes members of groups smaller than five.

^c^Research categories are not exclusive.

### Current biospecimen collection practices and resources

The majority (71.4%) of the respondents indicated never discontinuing research due to lack of specimens, while 30 respondents (26.8% of total) reported discontinuation (Table 3). Almost half of the respondents (n = 51, 45.5%) reported having collected human tissues/biospecimens for their research work within the past 5 years (Table 3). Of those working with human biospecimens, the most frequently reported collected samples were biopsies and blood samples, by 39 and 37 participants respectively. Regarding the ethnic diversity of tissues collected, 20 respondents reported that <19% of their tissue collections were from diverse donors, while 11 respondents were not aware of the ethnicity of donors for their specimen collections. Many researchers reported knowing the gender and age of their donors whereas less than a quarter reported knowing the race (n = 26) or ethnicity (n = 25). Slightly less than half of the researchers (n = 53) responded to the questions about informed consent; of those who responded, 29 (25.9%) used a specific purpose informed consent (i.e., for use only for the specific analyses described in the informed consent statement).

**Table 3:** Current biospecimen collection practices and resources.

Practice or Resource	Sub-category	# of participants (% respondents)	% of 112 total participants
Discontinued research in past 10 years due to insufficient biospecimens (# of respondents who answered this question = 105)		30 (28.6^a^)	26.8
Collected biospecimens within the past 5 years (# of respondents who answered this question = 112)		51 (45.5)	45.5
Types of tissues being collected (# of respondents who answered this question = 51)	biopsies	39 (76.5)	34.8
	blood	37 (72.5)	33.0
	urine	10 (19.6)	8.9
	buccal swabs/saliva	10 (19.6)	8.9
	hair	1 (2.0)	0.1
Ethnic diversity of biospecimen collection (# of respondents who answered this question = 51)	0–9%	8 (15.7)	7.1
	10–19%	12 (23.5)	10.7
	20–39%	8 (15.7)	7.1
	>40%	12 (23.5)	10.7
	ethnicity unknown	11 (21.6)	9.8
Knowledge of donor	gender (49^b^)	47 (92.2)	42.0
	age (45)	43 (84.3)	38.4
	ethnicity (34)	25 (49.0)	22.3
	race (36)	26 (51.0)	23.2
	medical history (45)	37 (72.5)	33.0
	quality of life (25)	8 (15.7)	7.1
Type of consent for biospecimen collection (# of respondents who answered this question = 53)	any research purpose	24 (45.3)	21.4
	specific research only	29 (54.7)	25.9

^a^Sub-category percentage calculated as number of participants with sub-category response/number of respondents for that sub-category, eg. 28.6 = 30/105 x 100

^b^number of respondents who answered this question

### Attitudes to sharing biospecimens and participating in a virtual national biorepository

Overall, the majority of respondents (n = 99, 88.4% of total) agreed with the NIH Resource Sharing Plan and indicated they considered “human biospecimens” as resources that should be shared (Table 4). More than half of 78 respondents who answered the questions about using a virtual biorepository would be likely or very likely (n = 47, 42.0% of total) to obtain specimens using such a biorepository with similar numbers of respondents (n = 50, 44.6% of total) likely or very likely to share data about their biospecimens in a virtual national biorepository. More respondents (n = 92) reported their preferences for the format for a virtual national biorepository; most of them (n = 42, 37.5% of total) selected a login-access only database. Similar numbers preferred a curated-access only database (n = 23) versus a publicly available database (n = 21).

**Table 4:** Perceptions and attitudes about a virtual biorepository expressed by survey respondents.

Characteristic	Sub-characteristic	# of participants (% respondents)	% of 112 total participants
Agree that human biospecimens are resources covered by “NIH Resources Sharing Plan” (# of respondents who answered this question = 109)		99 (90.8^a^)	88.4
Likelihood of obtaining samples from a virtual biorepository (# of respondents who answered this question = 78)	very likely/likely	47 (60.3)	42.0
	neutral	16 (20.5)	14.3
	unlikely/very unlikely	15 (19.3)	13.4
Likelihood of sharing samples from a virtual biorepository (# of respondents who answered this question = 78)	very likely/likely	50 (64.1)	44.6
	neutral	15 (19.2)	13.4
	unlikely/very unlikely	13 (16.7)	11.6
Preferred format for a virtual biorepository database (# of respondents who answered this question = 92)	log in access only	42 (45.7)	37.5
	curated access only	23 (25.0)	20.5
	publicly available	21 (22.8)	18.7
	none	6 (6.5)	5.4
Biospecimen data types to be shared on a virtual biorepository (# of respondents who answered this question = 112)	number of specimens	74 (62.2)	62.2
	tissue/sample types	72 (60.5)	60.5
	sample collection methods	66 (55.5)	55.5
	clinical diagnosis of donors	64 (53.8)	53.8
	age of donors	64 (53.8)	53.8
	gender of donors	60 (50.4)	50.4
	ethnicity of donors	53 (44.5)	44.5
Researcher information to be shared on a virtual biorepository (# of respondents who answered this question = 112)	name of institution	87 (73.1)	73.1
	link to publications	73 (61.3)	61.3
	source of funding	61 (51.3)	51.3
	grant abstract	51 (42.9)	42.9
Aware of TCGA (# of respondents who answered this question = 77)		52 (67.5)	46.4
Using TCGA in research (# of respondents who answered this question = 110)		23 (20.9)	20.5

^a^Sub-characteristic percentage calculated as number of participants with sub-characteristic/number of respondents for that sub-characteristic, eg. 99/109 x 100 = 90.8%

All of the respondents (n = 112) indicated the different types of information they would agree to share/publish in an NIH-funded database (Table 4). The most frequently reported information likely to be published were the number of specimens and the tissue sample types in the biospecimen/data collection (by 62.2% and 60.5% of the respondents respectively). At least half of the researchers would agree to publish methods of sample collection and clinical diagnosis of specimen donors, as well as their age and gender. The majority of respondents (73.1%) reported agreeing to publish the name of their institution; as well as links for publications and research (61.3%), and source of funding (51.3%) (Table 4). However, slightly less than half of the respondents (n = 53, 44.5%) would be willing to report donor ethnicity.

Only 52 researchers (46.4% of total) in the region are aware of TCGA (Table 4) and fewer (n = 23, 20.5% of total) reported using this tool in their research programs. This survey did not measure whether these researchers were looking at gene expression studies, or genome perturbations and cancer. Researchers conducting genomic studies would be expected to be aware of TCGA for use in their research.

Researchers’ willingness to share information in a virtual biorepository varied based on their self-reported research category (basic, translational, clinical or epidemiological as defined [70]). All of the participants provided research category data. As Table 5 shows, basic researchers were significantly more likely to share specimen information on a virtual national biorepository compared with non-basic researchers (4.05 vs. 2.24, p < 0.01), whereas translational researchers were significantly less likely to share specimen information than non-translational researchers (2.10 vs 3.85; p < 0.01). Basic researchers were less likely to share grant information than non-basic researchers (1.40 vs. 2.43, p < 0.01), whereas clinical researchers were more likely to share grant information than non-clinical researchers (2.11 vs. 1.33, p < 0.05). No differences across researcher types were identified for donor information.

**Table 5:** Contrasts comparing different type of researchers against all others in their willingness to share different types of information (scale means).

	Researcher type^a^											
	Basic			Translational			Clinical			Epidemiology		
Scale^b^	Basic	others	*p*	Trans	others	*p*	Clin	others	*p*	Epi	others	*p*
SI. Specimen information	4.05	2.24	0.01	2.10	3.85	0.01	2.79	4.50	*ns*	2.88	4.60	*ns*
SII. Donor information	1.94	2.16	*ns*	2.29	1.92	*ns*	2.02	2.00	*ns*	1.98	2.40	*ns*
SIII. Grant information	1.40	2.43	0.01	2.15	1.91	*ns*	2.11	1.33	0.05	2.02	2.50	*ns*

^a^As defined in [70]: basic researchers are interested in general knowledge and an understanding of nature and its laws; translational researchers apply basic science discoveries to the treatment or prevention of human disease; clinical researchers work with human subjects or on materials of human origin for which an investigator directly interacts with human subjects.

^b^Specific items comprising each scale are defined in Table 1.

### Gender differences in biospecimen sharing practices

We found few differences regarding attitudes and behavioral intentions with respect to any socio-demographic characteristic of respondents including ethnicity, gender, age or state in which respondents were based. However, there were significant gender differences in response to the question, “do you use a general research purpose informed consent or do you use a specific purpose informed consent” (p < 0.05). Among the male researchers who answered this question 18 (54.5%) used the general purpose and 15 (45.5%) used the specific purpose informed consents with donors. In contrast, female researchers were significantly more likely to collect biospecimens from donors using a specific purpose informed consent (n = 14, 73.7%) than the general-purpose consent (n = 5, 26.3%) with donors.

### Summary of qualitative data

The descriptive summaries of the qualitative data, captured in the open-ended survey questions, provided a richer understanding of the issues impacting the implementation of an effective virtual national biorepository. Such information is particularly relevant to Region 4, a region with high population diversity and cancer health disparities. Below we present the most commonly reported themes for each of the four open ended questions on the survey and we list the frequency of these common themes based on the ethnicity of the respondent. The full list of themes generated by the responses for each of the four questions is presented in S1 Table, S2 Table, S3 Table and S4 Table, as well as a thematic description of all responses (S5 Table). In each of the summary tables presented for the four open-ended questions (Tables 6–9) we list the top six themes, and report the frequency (%) of their occurrence relative to the total number of participants (n = 112) and the frequency (%) to the number of participants who answered the specific question. Table 6 summarizes the responses from 66 participants, and Tables 7–9 from 28, 46 and 32 respondents respectively. Providing both scales allows the relative importance of these themes to be fully appreciated.

**Table 6:** Perceived barriers to implementation of a virtual national biorepository.

Themes	% of 112 total participants	All (66^a^)	Males (32)	Females (34)	NHW^b^ (44)	Minority (18)
Ethical barriers	22.3	25 (37.9^c^)	10 (31.3)	14 (41.2)	16 (36.4)	8 (44.4)
Legal barriers	16.1	18 (27.3)	11 (34.4)	6 (17.6)	12 (27.3)	5 (27.8)
Lack of standardized procedures	12.5	14 (21.2)	6 (18.8)	6 (17.6)	10 (22.7)	2 (11.1)
Lack of data or sample sharing	11.6	13 (19.7)	8 (25.0)	5 (14.7)	8 (18.2)	5 (27.8)
Funding barriers	11.6	13 (19.7)	7 (21.9)	4 (11.8)	9 (20.5)	2 (11.1)
Sample issues	10.7	12 (18.2)	6 (18.8)	6 (17.6)	10 (22.7)	2 (11.1)

^a^number of participants who answered these survey questions.

^b^NHW, non-Hispanic white

^c^Percentage of participants who ranked this barrier calculated based on demographic category of respondent, eg. 37.9% = 25/66 x 100

**Table 7:** Perceived reasons individuals refuse to donate specimens.

Themes	% of 112 total participants	All (28^a^)	Males (13)	Females (15)	NHW^b^ (23)	Minority (4)
Inconvenience	11.6	13 (46.4^c^)	5 (38.5)	8 (53.3)	9 (39.1)	4 (100.0)
Health concerns	10.7	12 (42.9)	5 (38.5)	6 (40.0)	10 (43.5)	1 (25.0)
Recruitment barriers	8.0	9 (32.1)	6 (46.2)	2 (13.3)	6 (26.1)	2 (50.0)
Privacy and security barriers	8.0	9 (32.1)	5 (38.5)	4 (26.7)	9 (39.1)	0 (0)
Misuse of personal information	7.1	8 (28.6)	3 (23.1)	5 (33.3)	6 (26.1)	2 (50.0)
Distrust in research/health care system	6.2	7 (25.0)	1 (7.7)	5 (33.3)	4 (17.4)	2 (50.0)

^a^number of participants who answered these survey questions.

^b^NHW, non-Hispanic white

^c^Percentage of participants who ranked this barrier calculated based on demographic category of respondent, eg. 37.9% = 25/66 x 100

**Table 8:** Researcher requirements for collaborating and sharing data.

Themes	% of 112 total participants	All (46^a^)	Males (22)	Females (24)	NHW^b^ (35)	Minority (10)
Collaboration and acknowledgment	12.5	14 (30.4^c^)	5 (22.7)	8 (33.3)	12 (34.3)	1 (10.0)
Expertise in tissue research	11.6	13 (28.3)	6 (27.3)	7 (29.2)	11 (31.4)	2 (20.0)
Compliance with institutional and federal policies	8.9	10 (21.7)	5 (22.7)	4 (16.7)	8 (22.9)	1 (10)
Data sharing policies	6.2	7 (15.2)	2 (9.1)	5 (20.8)	5 (14.3)	2 (20.0)
Preservation of resources	4.5	5 (10.9)	2 (9.1)	3 (12.5)	3 (8.6)	2 (20.0)

^a^number of participants who answered these survey questions.

^b^NHW, non-Hispanic white

^c^Percentage of participants who ranked this barrier calculated based on demographic category of respondent, eg. 37.9% = 25/66 x 100

**Table 9:** Researcher concerns if unwilling to share specimens.

Themes	% of 112 total participants	All (32^a^)	Males (17)	Females (15)	NHW^b^ (24)	Minority (7)
Plausibility of research	10.7	12 (37.5^c^)	8 (47.1)	3 (20.0)	8 (33.3)	3 (42.9)
Intellectual property rights	6.2	7 (21.9)	2 (11.8)	5 (33.3)	3 (12.5)	4 (57.1)
IRB Concerns	6.2	7 (21.9)	2 (11.8)	4 (26.7)	3 (12.5)	3 (42.9)
Costs/lack of reimbursements	6.2	7 (21.9)	2 (11.8)	5 (33.3)	7 (29.2)	0 (0)
Sample issues	6.2	7 (21.9)	4 (23.6)	3 (20.0)	7 (29.2)	0 (0)
Lack of expertise in tissue research	4.5	5 (15.6)	0 (0.0)	4 (26.7)	3 (12.5)	1 (14.3)

^a^number of participants who answered these survey questions.

^b^NHW, non-Hispanic white

^c^Percentage of participants who ranked this barrier calculated based on demographic category of respondent, eg. 37.9% = 25/66 x 100

#### Barriers to implementation of a virtual national biorepository

In the free response section of the survey, participants identified the major barriers to implementation of a virtual national biorepository; 66 researchers identified 17 thematic barriers (S1 Table). The top six thematic barriers reported by the researchers are listed in Table 6. These themes were “*ethical issues*”, “*legal barriers*”, “*lack of standardized procedures*”, “*lack of sharing*”, “*funding*”, and “*sample issues*”. All researcher groups listed ethical issues and legal barriers as top barriers to implementing a virtual biorepository. Slightly less than a quarter of non-Hispanic white (NHW) researchers (n = 10) also reported lack of standardized procedures and sample issues as top barriers, whereas slightly more than a quarter of minority researchers (n = 5) listed lack of data/sample sharing as a top barrier. For the most part, male and female researchers reported common barriers (Table 6); ethical barriers were the most commonly cited barriers among female researchers, while male researchers listed legal barriers most frequently. Examples of responses and their assigned barrier theme are presented here:

*“I work with American Indian people of the Southern Plains*. *I would need permission from each tribe in order to store and share biospecimens from a national biorepository*. *They have cultural reasons*, *as well as historical mistrust*, *for not being amenable to sharing biospecimens*.*” (Ethical barrier)*

*“Our IRB limits our studies to very small samples of tissue*, *as not only will other investigators at our institution request the same samples*, *but also because the sample needs to be preserved for legal purposes*.*” (Legal barrier)*

*“I personally believe that a 'national' repository is a fantasy*.*” (General barrier)*

#### Perceived reasons individuals refuse to donate

Based on their experiences, respondents who collected human biospecimens were asked to identify the most common perceived reasons individuals refuse to donate specimens; 28 researchers responded to this question and identified a total of 12 reasons for patients refusing to donate specimens for research (S2 Table). The top six themes for perceived reasons individuals refuse to donate are listed in Table 7. These themes were “*inconvenience*”, “*health concerns*”, “*recruitment barriers*”, “*privacy and security issues*”, “*misuse of personal information*”, and “*distrust in the health care system”*. All researcher groups perceived inconvenience and health concerns for the donor as reasons for refusal to donate. Female researchers (n = 5) also listed potential for misuse of personal information and distrust in the health care system as top reasons for individuals refusing to donate specimens, while males (n = 6) reported recruitment barriers and privacy/security as other top reasons for refusal (Table 7). Among NHW researchers an equally important reason individuals refuse to donate included privacy and security issues, whereas none of the minority researchers listed this as a reason. Among minority researchers (n = 2), recruitment issues, misuse of personal information and distrust of the health care system were perceived to be likely reasons donors refused to donate. Examples of responses and their assigned concern theme are presented here:

*“Inconvenience of additional procedure (including pain*, *etc*.*)*, *not clear about what the purpose of the biospecimen collection*, *fear of unexpected consequence of donating*, *potential expense*.*” (Inconvenience)*

*“Risk of cutting needle biopsies (for cancer patients)*. *Aversion to needles (if a blood draw)” (Health concern)*

#### Researchers’ requirements for collaborating and sharing specimens

Respondents identified their requirements for collaborating and sharing specimens with other investigators; 46 respondents identified 11 requirements for collaborating (S3 Table). The top five themes for requirements reported by the study sample are listed in Table 8. These themes included “*collaboration and acknowledgment*”, “*expertise in tissue research*”, “*compliance with institutional and federal policies*”, “*sharing data*”, and “*preservation of resources*”. For the most part, the different groups similarly emphasized the need for collaboration/acknowledgment and expertise in tissue research as requirements. Males and NHW researchers emphasized compliance with institutional and federal policies as a requirement whereas women and minority researchers emphasized data sharing policies (Table 8). Examples of responses and their assigned concern theme are presented here:

*“Equal participation on analysis of data and interpretation of results*, *co-authorship on publications*, *selection authority for further data sharing*.*” (Collaboration and acknowledgment)*

*“Not providing specimens to individuals/organizations who do not understand our own regional issues or have limited to no experience in health sciences research or tissue research or who have no clinical experience/expertise*.*” (Expertise in tissue research)*

#### Major concerns if unwilling to share specimens

The survey sought to identify the major concerns of researchers who were unwilling to share specimens with other investigators; 32 respondents reported ten concerns (S4 Table). The top six themes for these concerns are reported in Table 9. The most commonly listed concerns were “*plausibility of research*”, “*intellectual property rights*”, “*legitimacy and lack of IRB approval*”, “*costs/reimbursements*”, “*sample issues*”, and “*lack of expertise in tissue research*”. All groups, with the exception of female researchers, reported that plausibility of research was a top concern for their unwillingness to share specimens. Female (n = 5) and minority researchers (n = 4) also emphasized intellectual property rights and IRB issues as top concerns. Both females and NHW researchers both highlighted cost concerns (Table 9). Examples of responses and their assigned concern theme are presented here:

*“Institute and PI's qualifications and expertise*. *Caliber of the science and purpose of the science*.*”* (Plausibility of Research)

*“The direct competition with my research program*.*”* (Intellectual Property Rights)

## Discussion

Overall, this study with Region 4 researchers discovered various barriers to obtaining sufficient biospecimen quantities, and more specifically to obtaining sufficient quantities of diverse biospecimens. Many of these barriers reported for the region were consistent with prior research, including donor refusal, inefficient annotation of biospecimens, and researchers’ unwillingness to share existing biospecimen collections. Addressing these barriers to accessing biospecimens is essential to combating cancer in general and cancer health disparities in particular.

### Barriers in biospecimen research

Perhaps the most important finding from this study documented the barrier to accessing diverse biospecimens from collections. The lack of access to sufficient biospecimens for research identified in this and other studies [14] is of great concern, since it can lead to delays or discontinuation of important research studies as indicated by the present study.

Past research has identified three major reasons why researchers may not have access to sufficient biospecimens. These include insufficient quantities and quality of needed biospecimens [11–13], inefficient processes for accessing biospecimens [3], and researchers’ unwillingness to share existing biospecimens [23,24]. This study assessed two of these reasons: insufficient quantities/quality of biospecimens and researchers’ unwillingness to share existing biospecimens. With regard to insufficient quantities, one contributing factor may be potential donors’ reluctance to donate biospecimens for research purposes. Although previous studies have examined potential donors’ reasons for refusing to donate [34,46,50], the present study was different in that it examined researcher’s ‘perceived’ reasons why individuals refuse to donate. Although researchers in Region 4 captured some similar reasons for refusing to donate from those self-reported by prospective donors in these other studies [34,46,50], we also found some distinct reasons reported by each group. A unique perceived reason for donor refusal reported by researchers consisted of recruitment barriers. These recruitment barriers spurred from poor researcher communication and misunderstanding of the research protocol by potential donors. A unique reason listed by prospective donors in previous studies not even mentioned by researchers is that they were not even aware of clinical trials and related opportunities for donating biospecimens [47,48,51]. Such differences indicate the importance of capturing both researcher and potential donor perspectives in order to gain a more comprehensive understanding of the communication necessary between researcher and donor to overcome barriers to donation and facilitate biospecimen donations from diverse populations.

In regards to researchers’ unwillingness to share existing biospecimens, researchers in this study reported several concerns consistent with previous studies assessing data or biospecimen sharing practices [15,54] including perceived plausibility of the research, intellectual property rights to biospecimen data, IRB concerns, costs and processing of samples. Overall, addressing concerns towards sharing biospecimens—like those identified above—and increasing positive attitudes toward a virtual biorepository are critical first steps toward promoting biospecimen and related data sharing. NIH and other federal agencies have attempted to develop and implement policies promoting data sharing, resource sharing and standardized procedures for collecting, storing, and distributing biospecimens. However, recent analysis of compliance with the NIH data sharing policy revealed a need for increased enforcement [17,49]. The hope is that proper enforcement of such policies will promote a culture supporting sharing of biospecimens and as well as related data and other resources.

In addition to general concerns about sharing biospecimens, a second problem, particularly for health disparities researchers, is limited availability of diverse tissue samples. Two major issues in this regard are insufficient collection of diverse samples and lack of annotation capturing such diversity within existing diverse biospecimen pools. To date, most biorepositories have low representation of samples from minority populations [45,49,50]—a fact consistent with what our survey respondents reported. Specifically, a substantial proportion of researchers in Region 4 who collected biospecimens in the past 5 years reported that only small proportions of their tissues originated from diverse populations (0% to 19% diversity in their collections). Similarly low levels were reported by biorepository administrators in BMaP region 3 [49]. These diversity numbers for Region 4 are surprisingly low considering that many states in the region have much higher population diversity rates. In the most diverse state, NM, Hispanics account for 46.3% of the population and Native Americans are 10.7% and in the least diverse state, OK, these two populations account for 21.8% (U.S. Census Bureau, 2010 Census http://factfinder.census.gov/faces/nav/jsf/pages/index.xhtml).

It is important to note that among the 51 survey respondents who reported the ethnic diversity of their collections, only 20 respondents reported having the recommended proportion of diversity among their biospecimen collections (20–40%). This lack of diversity may not be real but rather a reflection of poor annotation procedures. Specifically, over half of the biospecimen collectors in the ethnically diverse Region 4 reported that they did not engage in recording the race or ethnicity of the biospecimen donor. Such lack of standardization in annotating donor race/ethnicity limits all researchers’ capabilities for accessing diverse biospecimens, not just among the original collectors. Although Region 4 researcher networks may serve as potential sources of diverse biospecimens, greater efforts need to be directed toward improving annotation of important diversity data associated with these samples. Specifically, researchers working with diverse populations can take the lead in developing protocols that assure comprehensive annotation of such diversity in their biospecimen pools.

### Virtual data sharing: researcher preferences and requirements

One proposed strategy for addressing barriers to biospecimen research identified in past studies is the creation of a Virtual National Biorepository. One early attempt to create such a data portal was TCGA, an online tool providing researchers access to high quality genomic data of many distinct cancer types. Although the present study found that many respondents were aware of TCGA, 52 of the 77 question respondents; far fewer, only 23 of the 110 question respondents, were actually using this tool in support of their research. This finding may indicate that TCGA tools are not very intuitive which may lead to an aversion or reluctance to use them [71], or that these respondents are not conducting research that would utilize these tools. Perhaps enhanced training opportunities for TCGA tools, like the Region 4-G/BMaP Bioinformatics Workshop (Bioinformatics to Mine Human Biospecimens Data Workshop, Feb 2013, http://aces.nmsu.edu/bioinformatics/) will increase the number of researchers using these resources.

This study assessed researcher willingness to participate in a virtual biorepository, their preferences for the establishment of an effective virtual national biorepository, and perceived barriers for doing so. Importantly, this study demonstrated that researchers within the Region 4 network recognized the importance of sharing human biospecimens and acknowledged the NIH Resource Sharing plan. Furthermore, interest in participating in a virtual biorepository was strong among the survey respondents. Past research found a similar level of interest for participating in the national caHUB database [14]. These favorable attitudes toward a virtual national biorepository captured in this study are a positive finding but may not fully indicate whether a researcher would actually use such an online tool. This study further explored researcher preferences and barriers influencing the extent to which researchers will actively share or obtain biospecimens from a virtual national biorepository.

#### Researcher preferences and barriers for a virtual national biorepository

Study respondents reported a variety of preferences for the set up and operation of a virtual national biorepository. Some of these preferences were clearly related to human subjects protections. For example, researchers varied regarding which types of data to list in a virtual biorepository. Whereas a large proportion agreed that sample quantities and types should be shared, substantially fewer agreed to share data on donor race/ethnicity. One possible reason for this reluctance may be that researchers are concerned with donor confidentiality. This was suggested in the researchers’ perceived barriers to implementing a virtual biorepository, which highlighted ethical and legal issues as top barriers. These perceived barriers revolved around informed consent, cultural issues, tribal permissions, mistrust, and patient confidentiality/privacy issues. These concerns were also raised at a recent NCI workshop, and an important recommendation of this group was to “facilitate sharing of existing specimens” and “ease the burden of material transfer agreements” [6].

Underlying these various ethical and legal concerns was the potential for inappropriate, or unapproved research protocols with the biospecimens. This potential was further reflected in the type of consent process researchers used in their biospecimen collection process, general vs. specific. Our study suggests that researchers in Region 4, especially women, were more inclined toward a research-specific consent process. Although purely speculative, this reluctance toward a general consent process may reflect researchers working to protect against unapproved secondary uses of the samples or alienating donors who may perceive they might be stigmatized or economically disadvantaged by research outcomes. The Havasupai case documents the concern over stigmatization very clearly with the unapproved uses for schizophrenia and inbreeding [35,52]. Unfortunately, the preference for a research-specific consent over a general-purpose consent may prevent the use of remaining biospecimen samples to address additional questions ensuing from the original research [5]. It may also serve as a barrier to sharing biospecimens through a virtual national biorepository. The ethical and legal barriers discussed above may significantly hinder the establishment and use of a virtual biorepository and ultimately cancer health disparities research.

Practical issues regarding the operation and management of a biorepository, such as funding and lack of standardized procedures, were also highlighted in this study. With regard to standardized procedures, participants highlighted three major issues including quality control of tissue and data management (e.g., proper annotation), having standard operating procedures for biorepositories, and accurate informatics databases. Furthermore, the funding required for executing these standardized procedures was also emphasized (e.g., cost of obtaining, storing, annotating, and processing biospecimens).

Another issue focused on intellectual property rights to data and samples. As suggested in previous studies, this sense of perceived ownership may be related to the extensive resources involved in collecting and storing these biospecimens [24]. Specifically, biomedical researchers and investigators spend substantial time, money and effort collecting data and specimens for research and clinical trials [15]. These issues result in researchers developing a sense of ownership for the data and samples collected and wanting to preserve exclusive rights to data that has taken many years to produce.

One interesting finding in regards to data sharing preferences was identified across researcher types (basic, translational, clinical or epidemiological). Surprisingly, basic researchers were more willing to share specimen information (e.g., type, number and collection methods) than non-basic researchers. In contrast, translational researchers reported being less willing to share such specimen information relative to non-translational researchers. These contrasting patterns may be attributed to perceived differences in the utility of biospecimen information for their research purposes, with basic researchers placing greater value on making such information available. With regard to sharing grant information, basic researchers were less likely to share such information relative to non-basic researchers. Clinical researchers in contrast were more willing to share grant information (e.g., funding source, abstract, institution) than non-clinical researchers. Any reluctance to share grant information was a surprising response, as this information is already publicly available on both the publications that result from these research projects and at the funding agencies websites. Altogether, these differences in attitudes about sharing data may be attributed to the different roles each type of researcher assumes in the biospecimen collection and analysis process and balancing patient care with research objectives. Basic scientists are not likely to work directly with patients, while clinical scientists will have direct access to patients and have greater opportunities for biospecimen collection.

#### Researcher requirements for biospecimen and related data sharing

Researchers also reported their requirements for sharing their specimens and the associated data. The most frequently reported requirements included collaboration and acknowledgment by other researchers, expertise in tissue research, and compliance with institutional and federal policies. In regards to collaboration and acknowledgment, participants’ requirements included authorship rights, collegial interactions, participation in different research processes, and lack of competition with one’s own research program. Such requirements have been formerly reported as reasons for granting individual requests to share data [15,55]. The establishment of the BRIF policy is an example of efforts to provide appropriate acknowledgment and incentivize biospecimen and related data sharing among researchers [4,29–31].

In a meta-analysis of data sharing among largely non-biomedical researchers, “degree of control” was a recurrent theme [8]. This theme contained topics within the concerns described by the health disparities researchers polled here. Researchers also emphasized the plausibility of the proposed research and researcher expertise in working with clinical samples as important requirements for sharing biospecimen data and collaborating with other scientists. Once again, the issue of human subjects protections was highlighted in the requirement for complying with institutional and federal policies. These findings demonstrate the need for specific guidelines to assure biomedical researchers that their samples/data and intellectual property rights are protected.

This study confirmed researchers’ willingness to participate in a virtual national biorepository. Of those 78 participants who addressed this survey question, three times as many respondents, 47, were likely to use such a biorepository versus only 15, who were not likely to use such a tool. A majority of the total 112 participants in our study did not report agreement to be likely to use a virtual national biorepository. However, the richness of the qualitative responses from our respondents reflects sincere interest in improving collaboration for access to human biospecimens. Region 4 researchers listed clear specifications for establishing and using such a biorepository. These included specifications related to standardized procedures, funding, and protections of human subjects and intellectual property. Many of these requirements reported in our study are echoed in “best practices” described by the NCI among others [72].

## Conclusions and Future Directions

Major challenges exist in biospecimen research, including lack of access to biospecimens that are of high quality, well annotated, and from diverse populations. Whether these barriers are a function of donor refusal, inefficient annotation process, or researchers’ unwillingness to share specimens, they must be addressed, particularly if the goal is to fully understand the underlying biological factors contributing to cancer health disparities. A virtual national biorepository creates a central source where researchers can locate high quality, well-annotated biospecimens and their associated data. The good news is that a large majority of researchers report a willingness to use a virtual national biorepository for sharing and obtaining biospecimens and associated data, although they do specify certain preferences for its set up and use as well as requirements for collaboration.

It is essential to translate these preferences into actions in order to promote a strong culture of data sharing and collaborative research. Findings from this descriptive study, and similar comprehensive research [14,23,24], can help guide development and management strategies for a virtual national biorepository. Such strategies comprise creation of ideal infrastructures (e.g., security, access, personnel), standard operating policies/procedures (e.g., patient confidentiality, standardized annotation), best practices (e.g., biospecimen processing and distribution, informed consent, ethical/legal issues, quality control, intellectual property), and online database training opportunities (e.g. TCGA training) to encourage the use of online virtual biorepositories. Incorporating such strategies addressing researcher preferences for a virtual national biorepository can help safeguard against their perceived concerns and barriers and promote biospecimen and related data sharing and increase collaborative cancer health disparities research.

Finally, there is an imperative need to increase participation in biospecimen research by diverse populations, particularly in diverse regions such as Region 4, where the impact could be great. In this regard, efforts should be twofold. First, efforts should target researchers to encourage effective recruitment of diverse population through clear communication of research protocols to prospective donors. In addition, engaging researchers in collaborations with community-based researchers will help establish a donor pipeline. Direct community involvement should begin early in the process and include the development of the language for the informed consent process to ensure that maximal use of the samples for the broadest acceptable purposes is achieved. Others have presented this recommendation as well [35,73]. Second, efforts should focus on increasing the public’s awareness of biospecimen research through culturally relevant educational interventions that emphasize the importance of biospecimen research in addressing health disparities. This may help to encourage bio-specimen donation among racially and ethnically diverse populations.

Biospecimen and related data sharing by researchers working with populations from highly diverse regions is essential to leveraging knowledge and resources needed for the advancement of research on cancer health disparities. Although federal funding entities have initiated guidelines for data and resource sharing, future efforts should address researcher preferences addressed in this and previous studies in order to promote biospecimen research and collaboration to address cancer health disparities.

## Supporting Information

S1 Table Thematic list of barriers to implementation of a virtual national biorepository (n = 66). (PDF)

S2 Table Thematic list of perceived reasons individuals refuse to donate specimens (n = 28). (PDF)

S3 Table Thematic list of individual’s requirements for collaborating and sharing data (n = 46). (PDF)

S4 Table Thematic list of concerns if unwilling to share specimens (n = 32). (PDF)

S5 Table Specific participant responses grouped by thematic and topical categories. (PDF)

S1 Survey Tool Researchers' Attitudes to Participation in a Virtual Biorepository. (PDF)

S1 Dataset De-identified verbatim responses to open-ended survey questions. (XLSX)
