# Supplementary material for: To Share or Not to Share? A Survey of Biomedical Researchers in the U.S. Southwest, an Ethnically Diverse Region
Source: PLoS One. 2015 Sep 17;10(9):e0138239. doi: 10.1371/journal.pone.0138239 (PMC4574947; doi:10.1371/journal.pone.0138239)
Supplement: S1 Table — (PDF) [file pone.0138239.s001.pdf]

Supporting Information for

To Share or Not to Share?
A survey of biomedical researchers in the U.S. southwest, an ethnically diverse region

Mai H. Oushy[1], Rebecca Palacios[2], Alan E. C. Holden[3], Amelie G. Ramirez[3], Kipling J. Gallion[3], and Mary A. O'Connell[1,*]

[1]Plant and Environmental Sciences, New Mexico State University, Las Cruces, NM 88003 USA

[2]Public Health Sciences, New Mexico State University, Las Cruces, NM 88003 USA

[3]Institute for Health Promotion Research, University of Texas Health Science Center, San Antonio, TX 78229 USA

S1 Table. Thematic list of barriers to implementation of a virtual national biorepository (n=66)

**Themes**	**N (%)**
Ethical barriers	*25 (37.9)*
Legal barriers	*18 (27.3)*
Lack of standardized procedures	*14 (21.2)*
Lack of sharing	*13 (19.7)*
Funding barriers	*13 (19.7)*
Sample issues	*12 (18.2)*
Infrastructure	6 (9.1)
Bureaucracy	5 (7.6)
Recruitment	5 (7.6)
Loss of authority over samples	4 (6.1)
Intellectual property rights	2 (3.0)
Type of research	2 (3.0)
Regulatory barriers	2 (3.0)
Security issues	2 (3.0)
Data accuracy barriers	1 (1.5)
Lack of benefit	1 (1.5)
Availability of data to others	1 (1.5)
