# Supplementary material for: To Share or Not to Share? A Survey of Biomedical Researchers in the U.S. Southwest, an Ethnically Diverse Region
Source: PLoS One. 2015 Sep 17;10(9):e0138239. doi: 10.1371/journal.pone.0138239 (PMC4574947; doi:10.1371/journal.pone.0138239)
Supplement: S2 Table — (PDF) [file pone.0138239.s002.pdf]

Supporting Information for

To Share or Not to Share?
A survey of biomedical researchers in the U.S. southwest, an ethnically diverse region

Mai H. Oushy[1], Rebecca Palacios[2], Alan E. C. Holden[3], Amelie G. Ramirez[3], Kipling J. Gallion[3], and Mary A. O'Connell[1,*]

[1]Plant and Environmental Sciences, New Mexico State University, Las Cruces, NM 88003 USA

[2]Public Health Sciences, New Mexico State University, Las Cruces, NM 88003 USA

[3]Institute for Health Promotion Research, University of Texas Health Science Center, San Antonio, TX 78229 USA

S2 Table. Thematic list of perceived reasons individuals refuse to donate specimens (n=28)

**Themes**	**N (%)**
Inconvenience	*13 (46.4)*
Health concerns	*12 (42.9)*
Recruitment barriers	*9 (32.1)*
Privacy and security barriers	*9 (32.1)*
Misuse of personal information	*8 (28.6)*
Distrust in health care system	*7 (25.0)*
Cultural and religious barriers	4 (14.3)
Consent barriers	3 (10.7)
Lack of personal benefits	3 (10.7)
IRB barriers	2 (7.1)
Negative societal and insurance impact	2 (7.1)
No reason reported	1 (3.6)
