# Supplementary material for: To Share or Not to Share? A Survey of Biomedical Researchers in the U.S. Southwest, an Ethnically Diverse Region
Source: PLoS One. 2015 Sep 17;10(9):e0138239. doi: 10.1371/journal.pone.0138239 (PMC4574947; doi:10.1371/journal.pone.0138239)
Supplement: S3 Table — (PDF) [file pone.0138239.s003.pdf]

Supporting Information for

To Share or Not to Share?
A survey of biomedical researchers in the U.S. southwest, an ethnically diverse region

Mai H. Oushy[1], Rebecca Palacios[2], Alan E. C. Holden[3], Amelie G. Ramirez[3], Kipling J. Gallion[3], and Mary A. O'Connell[1,*]

[1]Plant and Environmental Sciences, New Mexico State University, Las Cruces, NM 88003 USA

[2]Public Health Sciences, New Mexico State University, Las Cruces, NM 88003 USA

[3]Institute for Health Promotion Research, University of Texas Health Science Center, San Antonio, TX 78229 USA

S3 Table. Thematic list of individual's requirements for collaborating and sharing data (n=46)

**Themes**	**N (%)**
Collaboration and acknowledgment	*14 (30.4)*
Expertise in tissue research	*13 (28.3)*
Compliance with institutional and federal policies	*10 (21.7)*
Sharing data	*7 (15.2)*
Preservation of resources	*5 (10.9)*
Transparency	4 (8.7)
Size and type of samples	4 (8.7)
Ethical issues	4 (8.7)
Federally funded research	3 (6.5)
No reason given	2 (4.3)
Reimbursement for costs of processing specimens	1 (2.2)
