# Supplementary material for: To Share or Not to Share? A Survey of Biomedical Researchers in the U.S. Southwest, an Ethnically Diverse Region
Source: PLoS One. 2015 Sep 17;10(9):e0138239. doi: 10.1371/journal.pone.0138239 (PMC4574947; doi:10.1371/journal.pone.0138239)
Supplement: S4 Table — (PDF) [file pone.0138239.s004.pdf]

Supporting Information for

To Share or Not to Share?
A survey of biomedical researchers in the U.S. southwest, an ethnically diverse region

Mai H. Oushy[1], Rebecca Palacios[2], Alan E. C. Holden[3], Amelie G. Ramirez[3],
Kipling J. Gallion[3], and Mary A. O'Connell[1,*]

[1]Plant and Environmental Sciences, New Mexico State University, Las Cruces, NM 88003 USA

[2]Public Health Sciences, New Mexico State University, Las Cruces, NM 88003 USA

[3]Institute for Health Promotion Research, University of Texas Health Science Center, San Antonio, TX 78229 USA

S4 Table. Thematic list of concerns if unwilling to share specimens (n=32)

**Themes**	**N (%)**
Plausibility of research	*12 (37.5)*
Intellectual property rights	*7 (21.9)*
Legitimacy and lack of IRB approval	*7 (21.9)*
Costs/reimbursements	*7 (21.9)*
Sample issues	*7 (21.9)*
Lack of expertise in tissue research	*5 (15.6)*
HIPPA barriers	2 (6.3)
Donor not notified of beneficial information	2 (6.3)
No benefit	1 (3.1)
No reason given	1(3.1)
