# Supplementary material for: To Share or Not to Share? A Survey of Biomedical Researchers in the U.S. Southwest, an Ethnically Diverse Region
Source: PLoS One. 2015 Sep 17;10(9):e0138239. doi: 10.1371/journal.pone.0138239 (PMC4574947; doi:10.1371/journal.pone.0138239)
Supplement: S5 Table — (PDF) [file pone.0138239.s005.pdf]

Supporting Information for

To Share or Not to Share?

A survey of biomedical researchers in the U.S. southwest, an ethnically diverse region

Mai H. Oushy[1], Rebecca Palacios[2], Alan E. C. Holden[3], Amelie G. Ramirez[3],

Kipling J. Gallion[3], and Mary A. O'Connell[1,*]

[1]Plant and Environmental Sciences, New Mexico State University, Las Cruces, NM 88003 USA

[2]Public Health Sciences, New Mexico State University, Las Cruces, NM 88003 USA

[3]Institute for Health Promotion Research, University of Texas Health Science Center, San Antonio, TX 78229 USA

S5 Table. Specific participant responses grouped by thematic and topical categories

**Barriers to implementation of a virtual national biorepository**	
**Theme/Topic**	**Illustrative quotes**
Ethical barriers<br>• Right/wrong conduct<br>• Being in accordance with rules or standards for right conduct<br>• Following accepted principles of right and wrong<br>• Fair<br>• Not doing harm to people or environment	▪ "…the concept of future uses possibly not specified during the original collection."<br>▪ "The Havasupai case has created enormous distrust of medical researchers among many of the western tribes of Native Americans, and has stirred up both governmental and cultural concerns."<br>▪ "Ethical issues"<br>▪ "Cultural factors: e.g., American Indians often want to be buried with the body intact."<br>▪ "…in a few previous attempts to participate in such a repository, we have never reached the level of insuring appropriate HIPAA protections just because the data has never been transferred."<br>▪ "Informed consent and the real ability to speak across cultures"<br>▪ "Consent process" (n=2)<br>▪ "Ethical barriers"(n = 6)<br>▪ "Another issue of course is informed consent."<br>▪ "a big problem as is the ethics regarding the information gathered - not sure how information/data gathered will be regulated."<br>▪ "ethical - if a donor donates for one purpose, they may not want their tissue used for a second unrelated purpose."<br>▪ "I work with American Indian people of the Southern Plains. I would need permission from each tribe in order to store and share biospecimens from a national biorepository. They have cultural reasons, as well as historical mistrust, for not being amenable to sharing biospecimens."<br>▪ "in an age in which the care of expensive diseases such as cancer are inherently resource -constrained, I would want to know about the uses to which human patient specimens are put and the motives of people who have access to them."<br>▪ "Ethical. We use human fetal tissues."<br>▪ "We have found that presentation of the consent information is critical."<br>▪ Ethical barriers"
Legal barriers<br>• Principles and regulations	▪ "Oversight- is everything being stored/handled properly."<br>▪ "Legal barriers"(n=7)<br>▪ "Mainly legal barriers."<br>▪ "Local IRB constraints"

established for a specific purpose<br>• Binding custom or practice of a community<br>• A rule of conduct or action prescribed or formally recognized as binding or enforced by a controlling authority	▪ "legal issues are the major barriers."<br>▪ "I understand that the law views biospecimens as no longer "belonging" to the person that gave them and this is very uncomfortable for many people."<br>▪ "Legal issues when dealing with specific ethnic groups."<br>▪ Our IRB limits our studies to very small samples of tissue, as not only will other investigators at our institution request the same samples, but also because the sample needs to be preserved for legal purposes."<br>▪ "IRB barriers"(n=2)<br>▪ "Legal issues when dealing with specific ethnic groups."<br>▪ "Obtaining adequate permissions from donors with all possibilities spelled out in terms of ultimate utilization of tissues"<br>▪ "I would need permission from each tribe in order to store and share biospecimens from a national biorepository."
Lack of standardization<br><br>• Lack of use of standardized and agreed upon procedures for biospecimen collection, storage, and annotation.	▪ "The major barriers (especially in our region) are the collection of bio/tissue samples themselves."<br>▪ "While it may be possible to work within a community to establish greater trust and interest in potential tissue-based or genetic research, the procedural barriers have definitely grown."<br>▪ "Consistency in pre-analytical handling of the samples. Clinical annotation that includes survival or outcome information."<br>▪ "organization."<br>▪ "annotation barriers"<br>▪ "Information related to concurrent diseases, therapies, or nutritional history."<br>▪ "For cancer populations, consistent pathological data on the molecular phenotype of the specimen."<br>▪ it is impossible to effectively de-identify the biospecimen (assuming that genetic information can be extracted from it).<br>▪ "General biobanks that take in all tissues and have no expertise or interest in the particular diseases they are collecting outside of just amassing as many different tissues as possible, often have poor quality tissues and inaccurate information (wrong diagnoses, minimal tumor involvement, necrotic samples, etc)."<br>▪ "The lack of standardization of protocols for tissue collection, processing, storage, retention, etc. are a major problem."<br>▪ "Informatics. The mechanism by which such repositories can build a database is very complex and in many instances it seems that these issues are not fully thought out or achievable when the concept is generated."<br>▪ "system/organization barriers"<br>▪ "Informatics Barriers"<br>▪ "Administrative organization of samples Ease of access to "sample" database Quality control of contributed samples."<br>▪ "Providing specimens in a biologically fresh and safe manner."<br>▪ "Proper sample identification"<br>▪ "Tissue handling and processing differences at different sites. Lack of standardized SOPs and information on the samples.
Lack of sharing<br><br>• Reluctance of researchers to share already existing biospecimens	▪ "sharing problems."<br>▪ "I would be very leery of tissues collected by others, and similarly protective of tissues and data that I collected where I know the processes and precious resources that went into collecting them. I personally believe that a 'national' repository is a fantasy."<br>▪ "competition among investigators."<br>▪ "Willingness to share specimens if requested."<br>▪ "data sharing decisions."

and/or biospecimens data with other researchers and/or institutions.	▪ "competition"<br>▪ "I collect biospecimens from patients with melanoma. The only other people who would want these are melanoma researchers and I am highly collaborative and we work out methods for sharing. I would not like to just give samples to anyone."<br>▪ "In general, a fear to be scooped by others potentially using one's data."<br>▪ "Having buy in"<br>▪ "Pathology labs/departments are unwilling to release tissues."<br>▪ "Sharing with investigators"<br>▪ "Big power big share, no power no share!"
Funding barriers<br><br>• Barriers related to costs of acquiring, processing, annotating and storing biospecimens appropriately. In addition to costs of the biospecimen sharing process.	▪ "If the NIH does not specifically pay for the procurement of biospecimens, and if other (institutional) funds are used for this purpose, then the NIH has no business in asking for these resources to be shared. How likely would it be for me to obtain funding if I put biobanking personnel into my grant budget? (Not very likely.)<br>▪ "cost of obtaining, storing and processing specimen, plus the cost of getting the information updated in a database."<br>▪ "Funding to support initiation and ongoing support."<br>▪ "Biospecimens such as buccal swabs could be shared if we had funding to conduct WGA, and sufficient staff to send it outside."<br>▪ "funding barriers"<br>▪ "lack of support for cataloguing updating and distribution of clinical samples for research.<br>▪ "financing"<br>▪ "Cost of preparation for sharing specimens."<br>▪ "Sufficient funding to accomplish biobanking, annotation and translational research."
Sample issues<br><br>• Barriers regarding the quality, quantity, availability, and access to biospecimen tissues.	▪ "Quality: I have observed that when biobankers have a vested interest in the disease type that they are banking (either clinical or research interest), the quality of the tissues is extremely high and accompanying information is accurate and sophisticated. General biobanks that take in all tissues and have no expertise or interest in the particular diseases they are collecting outside of just amassing as many different tissues as possible, often have poor quality tissues and inaccurate information (wrong diagnoses, minimal tumor involvement, necrotic samples, etc)."<br>▪ "the ability to represent the view of the populations from whom the specimens were collected."<br>▪ "It is very hard to get a large number of sample to examine."<br>▪ "Obtaining specimens."<br>▪ "Sequential patient samples are often needed but usually not available."<br>▪ "that samples are what they claim to be."<br>▪ "Local use of the discarded or biopsied tissue so that none is left to share or repose."
**Researcher's perceived reasons individuals refuse to donate samples**	
**Theme/Topic**	**Illustrative quotes**
Inconvenience<br><br>• Issues regarding tissue collection causing difficulty or trouble to the researcher.	▪ "Inadequate collection methods; 4) Inconvenience."<br>▪ "Inconvenience of coming in for sequential donations if required by the protocol."<br>▪ "(1) depending on how the tissue is collected, the request for an extra biopsy (2) lack of time."<br>▪ "no time!"<br>▪ "Inconvenience."<br>▪ "Time/hassle."<br>▪ "I have not had prohibitive refusal, however the burden of proper sample collection (identifying, recruiting, consenting, then collecting from

	children and parents) has seriously delayed my research."<br>▪ "(1) Additional procedure involved. (2) Additional visits involved for procedures."
Health concerns<br><br>• Health barriers causing discomfort and trouble to patients/participant s.	▪ "health concerns (ie, poor health due to disease)"<br>▪ "Risk of cutting needle biopsies (for cancer patients). Adversion to needles (if a blood draw)."<br>▪ "Too invasive"<br>▪ "pain"<br>▪ "discomfort discomfort discomfort"<br>▪ "perception of increased risk"<br>▪ "Anxiety about their surgery leading them to forego all other considerations"<br>▪ "Distracted by their clinical issues."<br>▪ "too tired to consent"
Recruitment<br><br>• Issues regarding recruitment of patients/individuals to participate in clinical trials research projects.	▪ "not understanding the purpose of biobanking and research using banked specimens"<br>▪ "Inadequate education and poor communication"<br>▪ "failure of health team to enroll patients"<br>▪ "Lack of enthusiasm by clinical coordinators, surgeons in explaining collections."<br>▪ "Misunderstanding of protocol"<br>▪ "not clear about what the purpose of the biospecimen collection and fear of unexpected consequence of donating"<br>▪ "complexity or inability to understand consents"<br>▪ "Do not really understand how they will be used."
Privacy and security issues<br><br>• Issues regarding the privacy of individuals participating in clinical trials research.	▪ " Identity fear"<br>▪ "Privacy concerns"<br>▪ "confidentiality"<br>▪ "They fear invasion of privacy"<br>▪ "lack of security"<br>▪ "Privacy and confidentiality issues"
Misuse of personal information<br><br>• Participants' fear of their personal information be used by other parties, or used inappropriately to eventually cause them harm.	▪ "A few are concerned about "genetic testing" in general."<br>▪ "fear that genetic information will be used against them"<br>▪ "Concerns about being victimized by misconduct"<br>▪ "Concern that information will be used in appropriately"<br>▪ "fear of abuse"<br>▪ "They want to remain "in control" of the data that comes from them."
Distrust in research/ health care system<br><br>• Issues causing mistrust and frustration with the	▪ "frustration with clinical personnel and health care system"<br>▪ "distrust of the medical establishment"<br>▪ "Mistrust of research institutions"<br>▪ "Lack of trust"<br>▪ "Do not trust researcher"<br>▪ "Mistrust"

health care system and medical personnel.	▪ "medical mistrust"
**Requirements for collaborating and sharing data**	
**Theme/Topic**	**Illustrative quotes**
Collaboration and acknowledgment<br><br>• Requirements for researchers to work with one another jointly in research projects. And ensuring each individual researchers receives appropriate credit for his work.	▪ "Specimens are used only by Cancer Center members and their collaborators."<br>▪ "must be willing to share in authorship provided that PI is an active participant in the project."<br>▪ "collegial interaction"<br>▪ "SHARED DEVELOPMENT OF PUBLICATIONS, GRANTS, ETC"<br>▪ "That they not be engaged in research directly competing with our own research direction unless the work is undertaken collaboratively, i.e. shared authorship."<br>▪ "That we work together"<br>▪ "depending of type and number the samples I would like to be co-author in the publication"<br>▪ "equal participation on analysis of data and interpretation of results co-authorship on publications"<br>▪ "remain part of the process, and authorship depends on substantial contribution"<br>▪ "Authorship rights"<br>▪ "Acknowledgement, unless collaborative research is undertaken"<br>▪ "Collaboration not required unless I have a specific interested in the project."<br>▪ "Collaboration- if have intellectual input, not just for providing materials"
Expertise in tissue research<br><br>• The quality of the proposed research protocol, and having prior legitimate experience in clinical trials research.	▪ "Must have an excellent research hypothesis"<br>▪ "Demonstrated expertise in working with clinical samples"<br>▪ "importance/impact of research question"<br>▪ "Plausibility of research (adequate power, sound hypothesis) Novelty - usually the project should test a hypothesis not fully tested previously"<br>▪ "Good science."<br>▪ "that the proposed studies have merit."<br>▪ "as long as it is well organized"<br>▪ "Not providing specimens to individuals/organizations who have limited to no experience in health sciences research or tissue research or who have no clinical experience/expertise."<br>▪ "Scientifically meritorious study"<br>▪ "A clear understanding of the structure, labeling, access"
Compliance with institutional and federal policies<br><br>• Being in accordance with the policies of institutions, protocols, and /or IRB.	▪ "Must have peer reviewed funding and must have approval by internal oversight committee."<br>▪ "Institutional requirements Federal requirements"<br>▪ "has IRB (human subjects research) approval."<br>▪ "Federally approved research IRB approval"<br>▪ "Review and approval by our IRB and Scientific Merit Committee. "<br>▪ "MTA, IRB, legal and Tech transfer office review of project submitted by requesting PI/Institution."<br>▪ "That this will be approved based on federal and institutional guidelines and rules"
Data sharing policies<br><br>• Policies that need to be met so that	▪ "Data generated from specimens to be made public for other researchers."<br>▪ "selection authority for further data sharing"<br>▪ "If the research is supported by federal agencies, it should be available

researchers share data with other institutions/researchers.	to all.”<br>▪ “Maintaining local control.”<br>▪ “informed of results before publication,”<br>▪ “I want this to be web-based.”<br>▪ “that (raw) data stemming from the research conducted with the specimens be available to us”
Preservation of resources<br><br>• Ensuring that biospecimen samples are not wasted	▪ “Any good science project that (1) does not deplete the samples”<br>▪ “(4) In some instances, due to the rare nature of the patient's type of sample, we have asked to see the feasibility study showing that the investigators will be able to perform the experiments they describe on some unrelated tissues or cell lines.”<br>▪ “There needs to be a board to ascertain that studies will not be duplicating earlier work with limited amount of sample.”<br>▪ “Will not deplete tissue supply Will not require excessive resource (cost or personnel)”<br>▪ “That when possible some level of data sharing occurs, mainly to avoid repeated analyzes of the same specimens.”
Transparency<br><br>• openness, communication and accountability	▪ “personal association and satisfaction that the tissue will be used and handled correctly.”<br>▪ “Trust”<br>▪ “open process”<br>▪ “transparency”
**Concerns if unwilling to share**	
**Theme/Topic**	**Illustrative quotes**
Plausibility of research<br><br>• Research that is worthy of approval or acceptance; credible.	▪ “Poor study designs”<br>▪ “waste of tissue for unsound projects.”<br>▪ “Novelty - usually the project should test a hypothesis not fully tested previously”<br>▪ “Wastage of specimens”<br>▪ “Loss of specimen if shared physically”<br>▪ “sample depletion”<br>▪ “poorly organized.”<br>▪ “I have been approached by individuals who have not even bothered to understand the data and who it applied to - "could I just send them some DNA"? I find that poor.”<br>▪ “Do not exhaust tissue for non- definitive studies.”<br>▪ “Caliber of the science and purpose of the science.”<br>▪ “get it and waste it.”
Intellectual property rights<br><br>• Rights given to researchers over the possession of biospecimens, creations of research project. Having exclusive rights over the use of his/her results, as well as rules for sharing samples/data.	▪ “Retribution in manuscript and grant review process.”<br>▪ “Will not be able to collaborate”<br>▪ “lack of reciprocity”<br>▪ “Maintaining local control”<br>▪ “Direct competition with my research program.”<br>▪ “Lack of authorship”<br>▪ “intellectual property issues”
IRB concerns	▪ “approval by internal oversight committee”

• Barriers regarding IRB approval/rejections due to issues or concerns in the protocol of the clinical trials.	▪ "…the investigator does not have IRB approval."<br>▪ "WITHIN CONSENT DOCUMENT AGREEMENT"<br>▪ "Donors not protected"<br>▪ "Lack of administrative/institutional legitimacy from requesting institution"<br>▪ "That a human subjects protocol covers all the participants."
Costs/reimbursements<br><br>• Concerns regarding the costs of sharing samples with other researchers/instituti ons	▪ "The cost of withdrawing the samples would also need to be carefully evaluated to make "sharing" practical and sustainable."<br>▪ "time and effort lost"<br>▪ "NEED TO COMPLETE MY OWN ANALYTICAL WORK"<br>▪ "Costs involved"<br>▪ "We have a few buccal samples, but do not have funds to amplify them and make them available."<br>▪ "individuals/organizations need to understand our regional issues"<br>▪ Cost of collection, archiving,…and distribution costs"
Sample issues<br><br>• Concerns regarding the availability, access to, quality and quantity of biospecimen tissues to be shared	▪ "On the rare occasion that we have not accommodated a request, it has been because we do not have sufficient numbers of samples"<br>▪ "Not enough sample to do both our research and that of other labs"<br>▪ "Malignant tissues cannot be shared as per above. We have a few buccal samples,"<br>▪ "At present we need almost all of our specimens for our own research"<br>▪ "Usually not enough specimen is available to share, as I do not maintain a tissue bank."<br>▪ "Not enough tissue left to spare"<br>▪ "Amount of material available"
Lack of expertise in tissue research<br><br>• Concerns over the level of experience in clinical trials and tissue research by researchers requesting use of biospecimen tissues and/or data.	▪ "Specimens are used only by Cancer Center members and their collaborators"<br>▪ "The biggest problem with our institution's biobank is a lack of a populated clinical annotated data base. This is a barrier for withdrawing meaningful samples for our own investigators, and would be for anyone outside the institution as well."<br>▪ "misinterpretation of data results"<br>▪ "*individuals/organizations need to have experience in health sciences research or tissue research *individuals/organizations need to have clinical experience/expertise"<br>▪ "Institute and PI's qualifications and expertise."
