# Supplementary material for: To Share or Not to Share? A Survey of Biomedical Researchers in the U.S. Southwest, an Ethnically Diverse Region
Source: PLoS One. 2015 Sep 17;10(9):e0138239. doi: 10.1371/journal.pone.0138239 (PMC4574947; doi:10.1371/journal.pone.0138239)
Supplement: S1 Survey Tool — (PDF) [file pone.0138239.s006.pdf]

Region 4 Researchers' Attitudes to Participation in a Virtual Biorepository IV

Introduction

The National Cancer Institute Center to Reduce Cancer Health Disparities Region 4 Minority Biospecimen/Biobanking-Geographic Management Program (BMaP) is participating in a cross-regional biospecimen database pilot to facilitate collaborative biospecimen research in cancer health disparities.

We would like to assess the interests of researchers in Region 4 towards the virtual repository concept. Your input will help us identify all bases for resistance as well as willingness to share biospecimen information across the regions.

Your participation in the survey is VOLUNTARY. You may choose to answer specific questions or refuse to complete the questionnaire. It will take approximately 10-15 minutes to complete.

There is no identifiable information in the survey and your responses will not be tracked back to you. Your responses will be entered into a database and we will code your information with a survey number for organizational purposes.

If you have any questions pertaining to this research, you may contact:

Mary O'Connell
Principal Investigator
575-646-5172
moconnel@nmsu.edu

Mai Oushy
Co-Investigator
575-646-2115
moushy@nmsu.edu

If you have any questions pertaining to your rights as a research subject, you may contact the Office of the Vice President for Research at:
575-646-7177
ovpr@nmsu.edu

***1. By accepting to proceed you agree to participate in the survey.**

◯ Yes, I do wish to participate in the survey.

◯ No, I do not wish to participate in the survey.

2. What is your role in the research?

- ◯ PI
- ◯ Other

3. Do you work at a PhD granting university?

- ◯ Yes
- ◯ No

4. How do you classify your research work?

- ☐ Basic
- ☐ Translational
- ☐ Epidemiological
- ☐ Clinical

Other (please specify)

5. Within the past 5 years have you collected human tissues/biospecimens for your research?
-If NO, skip to Question 9-

- ◯ Yes
- ◯ No

6. What type(s) of human tissues are you collecting or working with?

- [ ] Blood
- [ ] Urine
- [ ] Biopsies
- [ ] Buccal swabs/Saliva
- [ ] Hair

7. What type of information do you know about the donors of the biospecimens you work with?

- [ ] Gender
- [ ] Age
- [ ] Ethnicity
- [ ] Race
- [ ] Medical History
- [ ] Quality of life

8. What percentage of your tissue collections are from diverse populations?

- ○ 0-10%
- ○ 10-20%
- ○ 20-40%
- ○ >40%
- ○ ethnicity unknown

9. If a virtual national biorepository was created,would you obtain samples from it for your research?

◯ Very likely ◯ Likely ◯ Neutral ◯ Unlikely ◯ Very unlikely

10. If a virtual national biorepository was created, would you share data about biospecimens you have collected?

◯ Very likely ◯ Likely ◯ Neutral ◯ Unlikely ◯ Very unlikely

11. Are you aware of The Cancer Genome Atlas (TCGA)?

◯ Yes
◯ No

12. Do you use The Cancer Genome Atlas (TCGA)?

◯ Yes
◯ No

13. Resource Sharing Plan
"NIH considers the sharing of unique research resources developed through NIH-sponsored research an important means to enhance the value and further the advancement of the
research."

Do you consider "human biospecimens" as resources that should be shared?

◯ Yes
◯ No

14. In your opinion what are the major barriers to implementation of a virtual national biorepository (security/legal/ethical/recruitment)

15. In the past ten years have you discontinued research direction because you were unable to obtain sufficient biospecimens?

- ◯ Yes
- ◯ No

16. If you have collected human biospecimens, in your experience what are the four most common reasons individuals refuse to donate specimens?

17. If you have collected human biospecimens, when you consent patients for tissue collection, usually is it for:

- ◯ Any research purposes
- ◯ A specific research only

18. Which virtual biorepository database would you prefer:

- ◯ Publicly available
- ◯ Log in-access only
- ◯ Curated-access only (Region 4 staff/NIH staff)
- ◯ None

19. Which of the following data about your tissue collection would you agree to publish on an NIH-funded Region 4 database
-select all that apply-

- ☐ Number of specimens
- ☐ Age of specimen donors
- ☐ Gender of specimen donors
- ☐ Ethnicity of specimen donors
- ☐ Clinical diagnosis of specimen donors
- ☐ Methods of sample collection
- ☐ Tissue/sample types

20. Which of the following data about yourself and your research would you agree to publish on an NIH-funded Region 4 database
-select all that apply-

- [ ] PI contact name and information
- [ ] Institution
- [ ] Link to publications
- [ ] Grant abstract
- [ ] Source of funding

21. What are your requirements for collaborating and sharing specimens with other investigators?

22. What are your major concerns if you are unwilling to share specimens?

23. How do you identify yourself?

- [ ] Hispanic/Latino
- [ ] Non Hispanic White
- [ ] Native American
- [ ] African American
- [ ] Asian

Other

24. Are you:

- ○ Male
- ○ Female

25. What is your age group?

- ◯ <30
- ◯ 30-40
- ◯ 40-50
- ◯ 50-60
- ◯ 60-70
- ◯ >70
